# LECAR: Location Estimation-Based Congestion-Aware Routing Protocol for Sparsely Deployed Energy-Efficient UAVs

**DOI:** 10.3390/s21217192

**Published:** 2021-10-29

**Authors:** Imtiaz Mahmud, You-Ze Cho

**Affiliations:** School of Electronic and Electrical Engineering, Kyungpook National University, Daegu 41566, Korea; imtiaz@knu.ac.kr

**Keywords:** DTN, DTN-based routing protocol, energy-efficient routing protocol, energy-efficient UAV, FANET, location estimation-based routing protocol, LECAR

## Abstract

Energy-efficient routing has become a critical issue for advanced energy-hungry unmanned aerial vehicles (UAVs). Routing in a flying ad hoc network is always challenging and becomes even more critical when a small number of UAVs must cover a large area. The routing protocols based on the delay-tolerant network (DTN) are best suited for such scenarios. However, traditional DTN-based routing protocols depend on data dissemination to offer a better packet delivery ratio, leading to congestion and excess transmissions, causing heavy and unnecessary energy consumption. We propose a location estimation-based congestion-aware routing protocol (LECAR) to balance these two issues. Considering outdated location information, LECAR takes advantage of the mobility model to estimate the current location of the destination. In addition, LECAR routes a packet by considering both the distance to destination and buffer occupancy of the neighboring UAVs. Simulation results show that LECAR could ensure both a high packet delivery ratio and low energy consumption. Moreover, LECAR could provide a minimal number of transmissions, while minimizing the number of copies per packet at a time.

## 1. Introduction

Unmanned aerial vehicles (UAVs) have been going through rapid technological advancements because of their broader applications, flexibility, cost-effectiveness, and, most significantly, lifesaving functionalities. In recent years, intense research has been taking place on the connectivity issues of a UAV system [1,2,3]. Especially in a multi-UAV system, we observe rapid topological changes due to the higher mobility of UAVs [4,5]. This problem has led to a new area of research: the flying ad hoc network (FANET) [6,7,8]. Unlike previous ad hoc networks, such as the mobile ad hoc network (MANET) [9] and vehicular ad hoc network (VANET) [10,11], FANET offers specific challenges, making it unique [6,7,8]. The UAVs move much faster than other standard vehicles, and this fast speed results in rapid changes in the network topology, leading to frequent link disconnections. The crisis deepens when the resources are limited, for example, when a limited number of UAVs need to cover a large area. In such a scenario, UAVs rarely come across each other, leaving a concise time window for communication purposes. Most current routing technologies fail to adapt and ensure successful communication in this situation.

Recently, considering such worse-case scenarios, several researchers have focused on store-carry-and-forward-based routing approaches (i.e., the delay-tolerant network (DTN) based approach) to ensure successful packet delivery [12,13,14,15,16,17,18]. In this type of routing protocol, the UAV stores data until it meets a suitable UAV to successfully forward that data to reach the destination. Most DTN-based approaches focus on disseminating the data as much as possible, following a flooding-based approach where the sender forwards the data to all its neighbors, then the neighbors forward it to their neighbors, and so on until it reaches the destination [12,13,14]. Consequently, this data flooding consumes a significant amount of network resources, leading to a network where the nodes are highly active to deal with the data. Moreover, multiple copies of the same packet exist in the network, causing high congestion in the buffer of almost all UAVs, leading to frequent packet drops. Although some recent research on DTN-based routing focuses on maintaining a small number of copies, this research is underdeveloped and poses several shortcomings [15]. The multiple copies generate many transmissions between the UAVs, which can become a significant concern as it keeps the resources busy dealing with the duplicate packets. Thus, this strategy consumes a substantial amount of energy.

On the other hand, UAVs often have a shortage of energy. Especially for small UAVs, energy shortage is a paramount concern. If the small UAVs must cover a larger area, they have minimal scope to allow any unnecessary energy consumption. In such a situation, if the routing protocol consumes excessive energy, then it becomes a significant problem. However, at the same time, it is vital to establish an efficient network where UAVs can successfully exchange the acquired information with the command-and-control station to fulfill the mission successfully, leading to a great bargain.

Although the FANET comprises enormous challenges, it also has its advantages. In general, while involved in a mission, UAVs cooperate among themselves. They usually follow a path-planning mechanism based on a map shared among themselves. Implementing a common logic known to every UAV; the UAVs choose their path from the map. They can also estimate/plan their consecutive moves from this path mapping (i.e., which direction a UAV takes). If location information for the other UAVs is present, a UAV can estimate the path of any other UAV by applying the same path-planning logic. Even though the location estimation might not be exact, it can provide the best idea about any other UAVs’ current and next possible locations. This information can be of great advantage for the routing protocols in solving the connectivity issues without losing much power.

In this paper, we propose a novel location estimation-based congestion-aware routing protocol (LECAR) that incorporates the location estimation functionality of a path-planning mechanism in making the routing decision. Based on the location map and path-planning mechanism, LECAR estimates the location of a UAV. Then, considering the estimated location, LECAR routes the packet to a neighbor UAV, which moves closer to the destination and has enough space in the buffer to handle the packets. The key contributions are listed below:

1. A novel, complete routing protocol is proposed that successfully takes advantage of the location estimation process to route the packets in the proper direction;
2. This method considers the buffer congestion state of the neighboring UAVs to avoid congestion and ensure successful data transmission;
3. The LECAR protocol forwards the packets following a single copy unicast fashion. Thus, it highly minimizes the power consumption by excess and unnecessary data transmission;
4. The LECAR protocol successfully achieves a high packet delivery ratio compared with the compared routing protocols, while ensuring the minimum number of copies per packet;
5. Moreover, LECAR consumes a significantly small amount of energy in comparison with the considered DTN-based routing protocols.

The rest of the paper is organized as follows. Section 2 summarizes the related work, and Section 3 briefly describes the considered problem scenario. Section 4 details the proposed routing scheme, and Section 5 evaluates the proposed LECAR routing protocol compared to the present routing protocols. Finally, Section 6 concludes the paper.

## 2. Related Work

In this section, we briefly describe the recent DTN-based routing protocols for FANET, which focus on sparsely populated network scenarios. We also discuss the existing key energy-efficient routing protocols for FANET scenarios.

Kuiper et al. [14] presented a DTN-based routing protocol for the FANET, combining geographic beacon-less routing with the DTN-based store-carry-and-forward principle. They forwarded packets based on a greedy algorithm governed by the pheromone repel model and location service. A UAV regularly broadcasts the data until it locates a suitable UAV that holds that packet for forwarding, consuming a significant amount of energy.

Another MANET-oriented DTN-based routing protocol, Spray and Wait [12], although achieving the highest packet delivery ratio, primarily disseminates data by broadcasting them to all neighbors in the vicinity. The neighbors broadcast the data to their neighbors, and so on; thus, the data reach the intended receiver. However, as its mechanism implies, it consumes considerable resources in terms of energy, memory, and number of transmissions. These issues make it highly inappropriate for FANETs.

Bujari et al. [15] proposed a routing protocol incorporating location information. They considered a scenario where the path is preplanned and known to every UAV. The UAVs consider this information in making routing decisions and achieve a moderately better packet delivery ratio. However, this type of scenario is not common. Instead, missions, such as reconnaissance or target tracking, often involve cases where the UAVs choose their path in real time based on the path-planning mechanism and mission objective.

Arafat et al. [19] combined the store-carry-and-forward-based routing approach with location-aided forwarding for the post-disaster operations of UAVs in their proposed LADTR routing protocol. They introduced the communication ferry UAVs, which physically carry the data to the destination or the next-hop relay. Additionally, they introduced a location prediction method based on the Guess-Markov model [20] and location data. However, this proposal mainly focuses on successful and timely delivery rather than energy-efficiency.

Oubbati et al. [21] proposed an energy-efficient routing protocol for FANETs. They considered the movement information and residual energy level of the UAVs and predicted sudden link breakage. Inspired by the AODV [22] link discovery process, the UAVs decide the routing paths based on the link breakage prediction, energy consumption, and degree of connectivity of the discovered paths. Although the focus was to find an energy-efficient routing solution for UAVs, they did not consider a sparsely populated scenario where the UAVs seldomly come across each other.

Shi et al. [23] proposed another routing protocol focusing on the energy-efficiency of the UAVs. The network is divided into several clusters. Among the member of a cluster, a cluster head is selected based on the energy level, degree of connectivity, and relative velocity. Intra-cluster communication is conducted through direct contact, whereas inter-cluster communication occurs only via the cluster head, considering that the cluster head has the highest energy. However, a major drawback is, because all the inter-cluster communication is tunneled through the cluster head, it soon runs out of energy and fails the strategy. Moreover, this solution does not consider sparsely populated scenarios of UAVs.

Khelifi et al. [24] proposed another cluster-based approach considering the energy-efficiency of UAVs. They used the received signal strength indication to calculate the positions of the undetermined UAVs. The cluster heads are elected based on a fuzzy-based localization algorithm. Nevertheless, the strategy generates a significant overhead during the formation of clusters and the election of cluster heads. Again, it only considers scenarios where a considerable number of UAVs are present.

Another cluster-based routing focusing energy-efficiency of UAVs has been proposed by Aadil et al. [25]. They focused on minimizing the overhead to reduce energy consumption. They considered dynamically adjustable communication range based on the separation distance among the communicating UAVs. The clusters are formed, and cluster heads are selected based on the degree of the neighborhoods. Nonetheless, this strategy considers a pre-planned mobility model that is highly uncommon in most FANET scenarios. Besides, it does not consider scenarios of a small number of UAVs covering a large area.

Table 1 compares the features among the existing major strategies with the proposed LECAR. Overall, the current DTN-based routing protocols fail to serve the purpose of energy-efficient routing in most cases. Additionally, the existing energy-efficient routing protocols do not consider sparsely populated network scenarios. Therefore, we are encouraged to propose LECAR, a location estimation-based routing protocol that can energy-efficiently work in sparsely populated scenarios where the paths are not predefined.

## 3. Problem Description

In this work, considering a complicated and real-world scenario where UAVs must move apart and occasionally obtain the communication scope, we consider a reconnaissance mission. Following our previous work presented in [26], we consider a mission where a small number of UAVs have the task to simultaneously search for targets in a large area while intermittently tracking the detected targets and avoiding detection by the targets. We also consider that the UAVs follow the mobility model proposed in [26]. Although we designed LECAR especially for the mobility model proposed in [26], the concept of LECAR can be easily adapted to any other mobility model.

**Figure 1:**
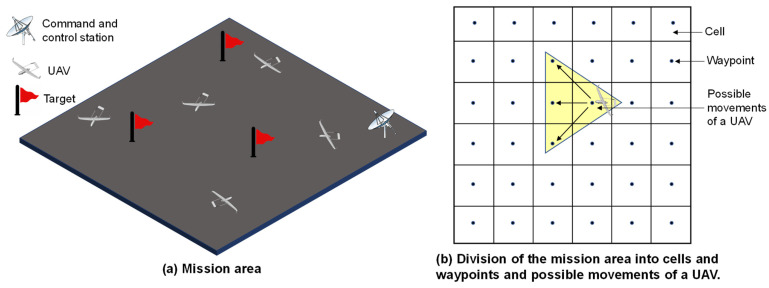
Illustration of the considered problem scenario: (**a**) the mission area and (**b**) the division of the mission area into cells and waypoints and the possible movements of a UAV.

In many mobility models, all UAVs use a shared map of the operational area for navigation, such as a probabilistic map, pheromone map, and others. The UAVs follow this map to determine their path. Following [26], we consider that the UAVs follow a pheromone map to select their real-time routes. The UAVs must continuously survey a 10km × 10 km area, and whenever they detect any target, they must follow it. The entire area is divided into small cells of 400m × 400 m, and we consider the center of each cell as a waypoint. Figure 1 illustrates the considerations. The UAVs are equipped with high-resolution cameras. Whenever a UAV passes over a waypoint, it implies that the UAV has successfully observed that cell. Based on the observation of a cell, the UAV leaves a pheromone value for that cell. Thus, each cell contains a pheromone value, and all cells together build a pheromone map. This pheromone map is periodically exchanged between UAVs so that they can obtain an update for the entire area and complete the mission cooperatively. We encourage the interested readers to read our previously proposed work in [26] for further details.

In addition, we consider that we have a limited number of UAVs to survey a large area. Therefore, the UAVs rarely encounter each other following the considered mobility model. Thus, UAVs have a concise time window to forward the packet to the destination. Whenever a UAV needs to send data to the command-and-control station or any other UAV, it might need to store that information in its buffer and forward that message whenever it encounters a suitable custodian. This data storage might lead to another problem of buffer overflow. For example, when a UAV sends a large amount of information, such as sensing data or high-resolution images, it requires ample space in the buffer to store the packets, which might lead to a buffer overflow. Therefore, to avoid packet drops, UAVs must be aware of the custodian’s buffer information. By custodian, we imply a neighboring UAV that can meet or travel near the destination and has enough memory in its buffer to store the message.

## 4. Location Estimation-Based Congestion-Aware Routing Protocol (LECAR)—Detailed Proposal

We propose a novel routing protocol, LECAR, that estimates UAVs’ location using the path planner and incorporates the results in the routing decision. As a DTN-based routing protocol, LECAR works in a single-copy unicast fashion. The protocol aims to ensure minimum resource usage and energy consumption, while maintaining a high packet delivery ratio.

In this section, we briefly describe LECAR in detail. We define the data tables that LECAR maintains for routing decisions, the packet format it shares with neighbors, and the hello message mechanism. Then, we describe the routing decision process of LECAR.

### 4.1. Defining Data Tables to Store the Necessary Information

To effectively select a suitable custodian, LECAR must keep a record of specific data. We briefly describe them in this section.

**Figure 2:**
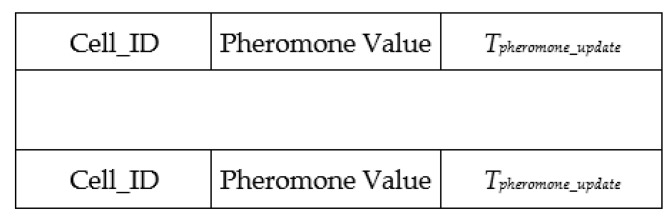
Data table format for storing a global pheromone map.

Figure 2 presents the global pheromone map table format that a UAV uses to keep the pheromone records for the mobility model. Each UAV uses this map to determine the path. *T_pheromone_update_* denotes the update time of the pheromone value for the corresponding cell.

**Figure 3:**
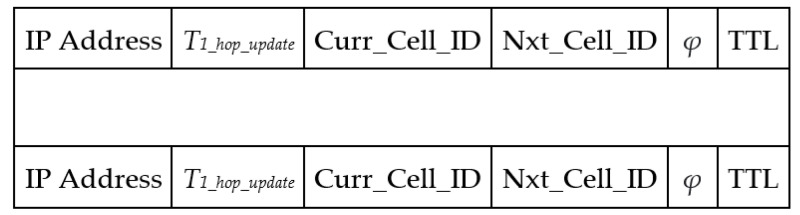
Data table format for storing the list of one-hop neighbors.

Figure 3 displays the one-hop neighbor table format. Each UAV maintains this table by exchanging hello messages. The notation *T_1_hop_update_*, Curr_Cell_ID, Nxt_Cell_ID, *φ*, and TTL denote the update time of the one-hop neighbor, current cell ID, next cell ID that the UAV will visit next, average buffer occupancy, and time to live of this entry. The TTL value is defined as follows:

(1) TTL=3×hello_interval.

**Figure 4:**
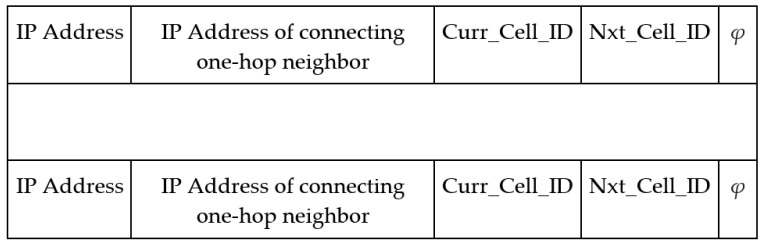
Data table format for storing the list of two-hop neighbors.

Figure 4 presents the two-hop neighbor table format. A UAV updates the two-hop neighbor entry whenever it receives a new list of neighbors from the one-hop neighbors. Moreover, a two-hop neighbor entry expires whenever the TTL of the corresponding one-hop neighbor expires.

**Figure 5:**
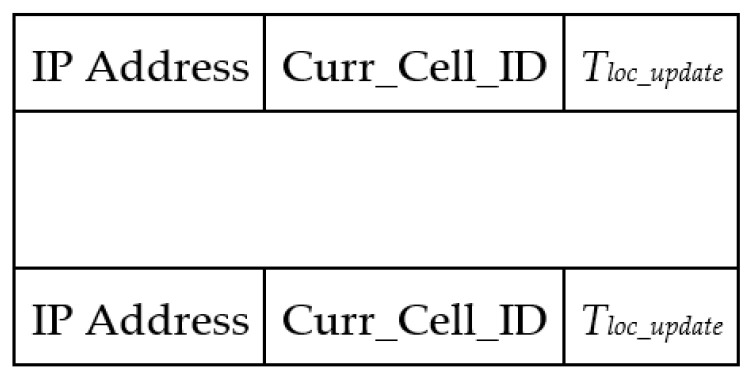
Data table format for storing the location information of UAVs.

Finally, Figure 5 illustrates the table format for storing the location information of the UAVs.

### 4.2. Defining Message Format and Interval

The UAVs must periodically exchange hello messages, global pheromone map, and location information for successful movement and communication.

#### 4.2.1. Hello Message Format

**Figure 6:**
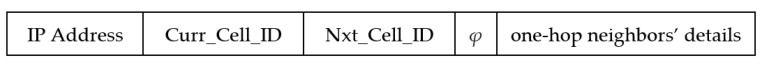
Hello message format.

**Figure 7:**
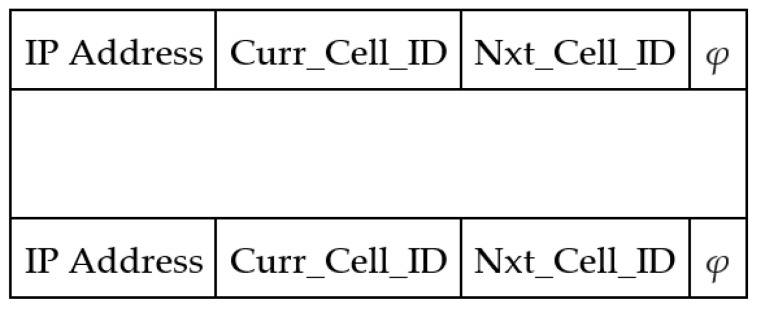
Message format for one-hop neighbor details in the hello message.

Figure 6 lists the hello message format details. The hello message also includes the one-hop neighbor details. Next, Figure 7 elaborates on the detailed information that a UAV sends for each one-hop neighbor.

#### 4.2.2. Hello Message Interval

We consider our previously proposed energy-efficient hello scheme with the instant hello message feedback mechanism for calculating the hello message interval [27]. Rather than sending hello messages after a fixed time interval, the hello interval is determined based on the speed of the UAV, airspace volume where UAVs are allowed to fly during the mission, and UAV transmission range. We encourage the interested readers to read details on the EE-hello scheme from [27].

#### 4.2.3. Global Pheromone Map and Location Information Message Format

**Figure 8:**
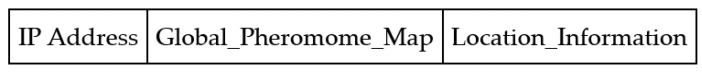
Global pheromone map and location information message format.

Figure 8 represents the format for the global pheromone map and location information message. The UAVs periodically exchange this message to update the pheromone map and location information of each other. Global_Pheromone_Map and Location_Information represents the global pheromone map and location information in Figure 2 and Figure 5, respectively.

#### 4.2.4. Global Pheromone Map and Location Information Message Interval

A UAV sends the global pheromone map and location information whenever a new one-hop neighbor is added to the one-hop neighbor table. In addition, the pheromone map and location information are updated and shared with the neighboring UAVs when a UAV reaches a waypoint.

#### 4.2.5. Update Mechanism

**Figure 9:**
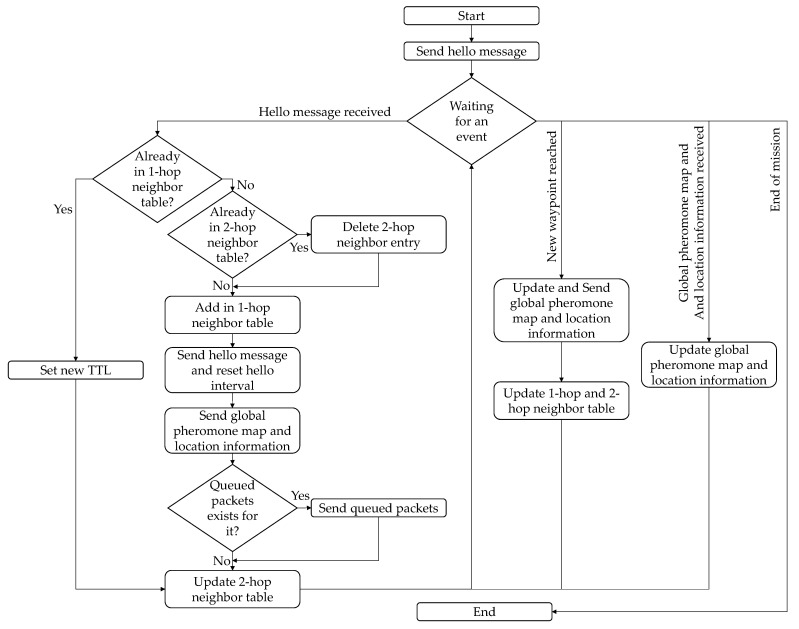
Flowchart with the update mechanism of the one- and two-hop neighbor data tables.

Upon reception of each hello message, the one- and two-hop neighbor tables are updated. The flowchart in Figure 9 summarizes the update mechanism. In addition, the global pheromone map and location information tables are updated each time the UAV reaches a waypoint or receives the global pheromone map and location information from a neighboring UAV. The UAV updates its global pheromone map and location tables using the entries with the latest *T_pheromone_update_* and *T_loc_update_*, respectively.

### 4.3. Routing Decision Process

We start this section with a simplified description of the routing process. As stated, every node has a global pheromone map and location table containing the tentative position of every UAV. Thus, a sender UAV knows its position, can easily learn the destination UAV position, and its one- and two-hop neighbor UAV positions. With the help of its path-planning mechanism, a sender can also estimate the next possible cell ID of the destination and its own. Concurrently, the sender considers its own and the one- and two-hop neighbor *φ* (i.e., average buffer occupancy). From this position information and *φ*, we propose that the sender selects a UAV from itself and its one-hop neighbors as a custodian. The custodian should have enough space to accommodate the packets in its buffer and is expected to be in the closest position with the destination in the near future. When a custodian UAV receives the packet, it follows the same procedures as the sender.

#### 4.3.1. Estimating the Location of the Destination

Without determining the exact location of a UAV, we intend to determine the cell ID where the UAV is hovering. We also consider that a UAV has a communication range that is twice the length of a cell. The UAV knows the other UAVs’ IP addresses and highest speed ranges. Moreover, UAVs share the global pheromone map and location information from the beginning of a mission. Thus, the pheromone map and location tables are populated from the beginning of a mission. The details on how the pheromone map is populated are in [26]. When a sender intends to send a data packet to the destination, the sender obtains the location of the destination from its location table. However, if we consider a scenario where the destination is far from the sender, we expect considerably old location information. In such a scenario, the sender uses its pheromone map and path-planning mechanism to estimate the destination’s current location/cell ID.

The sender calculates the number of waypoints (*n*) that the destination might have flown through after the last known location to estimate the current location of the destination, as follows:

(2) $$n=\frac{t_{passed}}{t_{s}}+1,$$

where *t_passed_* and *t_s_* denote the time passed after the update time of the last known location and the required time for a UAV to fly over a cell at its highest speed. Following its pheromone map and path-planning mechanism, the sender UAV estimates the flight path and current location/cell ID of the destination UAV.

#### 4.3.2. Calculating Distance

As mentioned, a sender UAV knows its location and the current and next location/cell ID of the one- and two-hop neighbors. The locations can be considered exact because the one- and two-hop neighbors’ location information is regularly shared through help messages. Following simple geometry, the sender UAV calculates the distance between any two UAVs:

(3) $$d_{ij}=\sqrt{{\left(x_{i}-x_{j}\right)}^{2}+{\left(y_{i}-y_{j}\right)}^{2}+{\left(z_{i}+z_{j}\right)}^{2}},$$

where *d_ij_* is the distance, and (*x_i_*, *y_i_*, *z_i_*) and (*x_j_*, *y_j_*, *z_j_*) are the coordinates of *UAV_i_* and *UAV_j_*, respectively.

Following Equation (3), the sender UAV calculates its current distance to the destination and the one- and two-hop neighbors’ current distances, represented by $d_{sd}^{1}$, $d_{n_{i}d}^{1}$, and $d_{n_{ij}d}^{1}$, respectively. Then, from the pheromone map and path-planning mechanism, the sender estimates which cell is next and then calculates its possible future distance to the destination, $d_{sd}^{2}$. In addition, the sender obtains the next cell ID for the one- and two-hop neighbors from the one- and two-hop neighbor table. Thus, it calculates the destination’s possible future distance from one- and two-hop neighbors ($d_{n_{i}d}^{2}$ and $d_{n_{ij}d}^{2}$, respectively).

#### 4.3.3. Calculating Normalized Distance

For the custodian selection, the sender calculates the normalized distance to incorporate the distance information with the congestion information successfully. Considering a two-hop neighbor, the sender UAV calculates the average distance ($avg\_d_{n_{ij}d}$) from the current and possible future distance between the considered two-hop neighbor and destination, as follows:

(4) $$avg\_d_{n_{ij}d}=\alpha \times d_{n_{ij}d}^{1}+(1-\alpha )\times d_{n_{ij}d}^{2},$$

where *α* is constant with a value of 0.5. Similarly, considering a one-hop neighbor, the sender calculates the average distance ($avg\_d_{n_{i}d}$) as follows:

(5) $$avg\_d_{n_{i}d}=\alpha \times d_{n_{i}d}^{1}+(1-\alpha )\times d_{n_{i}d}^{2}.$$

Generally, a one-hop neighbor connects to multiple two-hop neighbors. The sender creates pairs consisting of a two-hop neighbor and the one-hop neighbor through which the two-hop neighbor is connected to the sender. Such pairs are made for each of the two-hop neighbors. Then, the final average distance is calculated for each pair, as follows:

(6) $$F\_avg\_d_{n_{i}d}=\beta \times avg\_d_{n_{i}d}+(1-\beta )\times avg\_d_{n_{ij}d},$$

where *β* is a constant with a value of 0.5. The sender also calculates a $F\_avg\_d_{n_{i}d}$ for itself considering its current and future distance to the destination, as follows:

(7) $$F\_avg\_d_{sd}=\alpha \times d_{sd}^{1}+(1-\alpha )\times d_{sd}^{2}.$$

Finally, the sender considers the maximum possible distance (*max_distance*) between two UAVs in the considered mission area and calculates the normalized distance (Π) as follows:

(8) $$\Pi =\frac{F\_avg\_d_{sd}}{max\_distance}.$$

The sender calculates Π for itself and each pair of one- and two-hop neighbors.

#### 4.3.4. Calculating the Average Buffer Occupancy

Each UAV calculates its average buffer occupancy (*φ*) and shares it with neighboring UAVs through hello messages. Before sending a hello message, a UAV calculates its current buffer occupancy (*φ_c_*) as follows:

(9) $$\varphi_{c}=\frac{B_{current}}{B_{total}},$$

where *B_current_* and *B_total_* are the currently occupied buffer size and total buffer size, respectively. Then, it calculates the *φ* as follows:

(10) $$\varphi =\omega \times \varphi_{c}+(1-\omega )\times \varphi_{c},$$

where ω is a constant with a value of 0.6.

#### 4.3.5. Calculating the Forwarding Cost

To make the routing decision (i.e., to select the next custodian responsible for forwarding the packet), LECAR considers the estimated distance to the destination and the buffer occupancy. We demonstrated how to calculate the normalized distance and average buffer occupancy. Next, to combine these two, we introduced a parameter, the forwarding cost (*FC*), which is calculated as follows:

(11) $$\begin{array}{c} FC=(2-\Psi )\times \Pi +(\Psi -1)\times \varphi \\ \mathrm{where},\;\Psi =\left\{\begin{array}{l} e^{\varphi^{10}}\mathrm{when}\;\varphi <0.965\\ 2\;\;\;\;\;\mathrm{when}\;\varphi \geq 0.965 \end{array}\right. \end{array}$$

where *e* is an exponential constant and has a value of approximately 2.718. We incorporated this value following a previous study because buffer utilization is consistently low when the buffer occupancy is below 60% [28].

#### 4.3.6. Selecting a Custodian

As stated, the custodian can be the sender or any one-hop neighbor. The sender UAV calculates the *FC* value for itself and all pairs of one- and two-hop neighbors. Among the sender and one-hop neighbor of pairs, whichever has the lowest *FC* value becomes the custodian.

**Figure 10:**
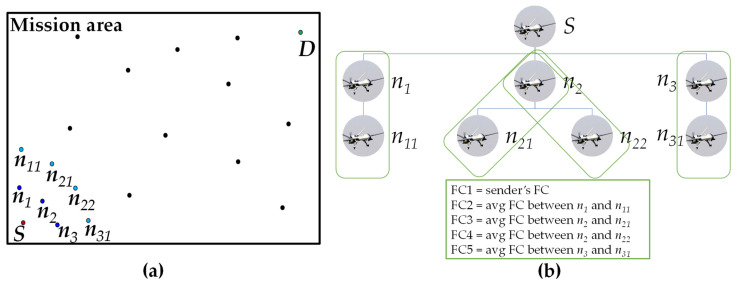
Simplified 2D example of *FC* calculation: (**a**) sample mission scenario and (**b**) sample *FC* calculation method.

In Figure 10, we present a simplified 2D representation of the concept of the *FC* calculation. We consider that sender *S* is connected to three one-hop neighbors *n*_1_, *n*_2_, and *n*_3_. The two-hop neighbor *n*_11_ is connected via *n*_1_, *n*_21_ and *n*_22_ are connected via *n*_2_, and *n*_32_ is connected via *n*_3_. Thus, there are four two-hop neighbors. Considering the one- and two-hop neighbors and the sender, five *FC* values are calculated. If the sender has the lowest *FC*, then the sender is the custodian. Conversely, if the *FC* value of any pair is the lowest, then the one-hop neighbor from that pair is the custodian.

#### 4.3.7. Acknowledgment

When the sender UAV sends a packet to the custodian, the custodian sends back an acknowledgment (ACK) indicating that it successfully received the packet and took custody of it. Upon receiving the ACK, the sender discards the packet, considering that the custodian is solely responsible for forwarding the packet further. Following the best-effort approach, the destination does not send an ACK to the sender; instead, the destination sends an ACK only to the immediate custodian. By implementing this ACK mechanism, we ensure the minimum number of packet copies remains in the network at a time.

#### 4.3.8. Hop Count

We include a hop count field in the header of the data packet. The hop count indicates how many UAVs the packet has traveled through during its journey from the sender to the destination. Whenever a UAV receives a packet, it increases the hop count number by one. It is initialized as one at the sender. Furthermore, we put the maximum limit for the hop count as the maximum number of UAVs participating in the mission. This maximum limit avoids infinite tries if the packet fails to reach the destination.

#### 4.3.9. Packet Forwarding Interval

When the UAV itself has the lowest *FC* value, the UAV waits for a packet forwarding interval (*P_interval_*) period and starts the packet forwarding process again. The packet forwarding process is described in the following subsection. The *P_interval_* primarily considers that the packet should be sent after traveling a certain distance and is defined as follows:

(12) $$P_{interval}=\frac{1}{v\times R_{rate}},$$

where *R_rate_* is the retry rate with a value of 0.004 m^–1^, indicating that the custodian tries to send the packet after traveling every 250 m.

#### 4.3.10. Packet Forwarding Process

Whenever a packet reaches a UAV, the UAV first checks for the destination address. If this UAV is the destination, it sends an ACK to the immediately previous custodian, and if it is not the destination, the UAV checks the packet’s hop count. If the hop count is more than the number of UAVs participating in the mission, the UAV discards the packet. Then, the UAV checks its one- and two-hop neighbor list for the destination. If the destination is available on that list, it forwards the packet to the destination. If the neighbor is not found in the one- and two-hop neighbor list, the UAV calculates the *FC* value for itself and the one- and two-hop neighbor pairs. Then, it forwards the packet to the one-hop neighbor that belongs to the pair with the lowest *FC* value. If the UAV has the lowest *FC* value, it waits for the *P_interval_* time and starts the forwarding process again. Algorithm 1 summarizes the packet forwarding process.
**Algorithm 1** The packet forwarding mechanism in LECAR**Upon reception of a packet or upon expiry of***P_interval_*:     **if**
*destinaion_address == my_address*
**then**            **send** ACK            **return**     **end if**     **if**
*current_hop_count > hop_count_max*
**then**            **discard** packet            **return**     **end if**     **for all** one-hop-neighbor *i*
**do**            **if**
*destination_address == one_hop_neighbor_i*
**then**                   **send packet to**
*one_hop_neighbor_i*                   **return**            **end if**            **for all** two-hop-neighbor-via-*one-hop-neighbor-i j*
**do**                   **if**
*destination_address == two_hop_neighbor_j*
**then**                         **send packet to**
*one_hop_neighbor_i*                         **return**                  **end if**            **end for**     **end for**     **calculate**
*FC_p_*⋯⋯⋯⋯⋯⋯⋯⋯⋯/* calculating FC for myself*/     **for all** one-hop-neighbor *i*
**do**            **calculate**
*FC_i_*            **for all** two-hop-neighbor-via-*one-hop-neighbor-i j*
**do**                  **calculate**
*FC_ij_*                  **calculate**
*FC_p_*⋯⋯⋯⋯/* calculating FC from for two-hop-neighbor *j* and                                    the corresponding one-hop-neighbor i*/            **end for**     **end for**     *temp_FC* = 0     **for all** FC *p*
**do**            **if**
*temp_FC < FC_p_*
**then**                  *temp_FC = FC_p_*                  *nest_custodian = address_of_FC_p_*            **end if**     **end for**     **if**
*my_address == address_of_FC_p_*
**then**            **queue packet until**
*P_interval_*     **else**            **send packet to**
*address_of_FC_p_*     **end if**

## 5. Performance Evaluation

Network Simulator 3.26 was used to evaluate the proposed routing protocol. The UAVs started from the southwest corner of the reconnaissance area. All experiments were repeated 30 times to obtain reasonable statistical confidence. The main simulation parameters are presented in Table 2. Moreover, we compared the performance of LECAR with some existing routing protocols: Spray and Wait [12] as a DTN-based routing protocol and LAROD-LoDiS [29] and GPSR [30] as hybrid routing protocols. We also implemented a modified version of GPSR so that the UAVs can store the packets in the buffer until they locate a suitable custodian. We call this protocol GPSR-Q. In addition, we implemented a modified version of LECAR and called location estimation-based routing (LER). The LER has all the functionality of LECAR, except it does not consider the buffer occupancy for selecting the custodian.

We compared LECAR with the considered routing protocols in terms of the packet delivery ratio, hop count per packet, number of copies per packet, number of transmissions per packet, delay per packet, total overhead, and total consumed energy. For all cases, we compared two buffer sizes: 25 and 50 MB. Each UAV generated 1 MB of data packets per minute during the experiment.

### 5.1. Performance Evaluation for the Packet Delivery Ratio

**Figure 11:**
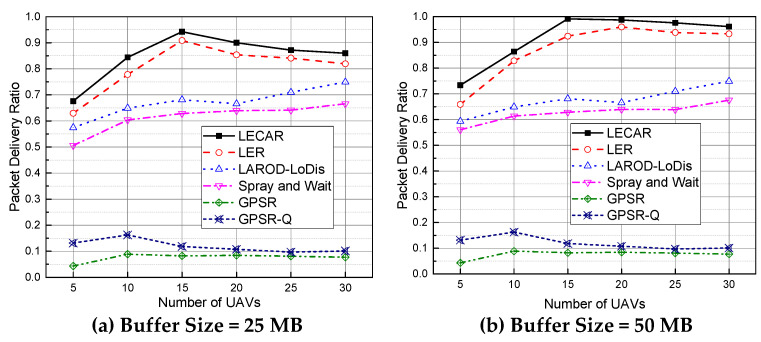
Performance comparison of routing protocols for the packet delivery ratio when the buffer size is (**a**) 25 MB and (**b**) 50 MB.

From Figure 11, LECAR achieves the highest packet delivery ratio compared with the considered routing protocols. The LER is the second-highest performer because it follows the same procedure as LECAR except for the buffer occupancy consideration. We believe that a lack of awareness of congestion leads to a performance decline in LER compared to LECAR. The GPSR performs the worst because it does not store the packet and tries to send it immediately, failing a maximum of times. The packets are delivered in GPSR only when the destination is a neighbor of the sender. GPSR-Q also lags far behind in the packet delivery ratio, indicating that if the routing is decided only based on the available location information, without considering a strategy for the sparse network scenario, the routing protocols suffer severely in achieving a considerable packet delivery ratio.

Spray and Wait performs far better than GPSR or GPSR-Q but slightly worse than LAROD-LoDiS. In Spray and Wait, the sender broadcasts packets to all neighbors, the neighbors broadcast to their neighbors, and so on until the packet reaches its destination. This spreading often causes significant congestion in the buffer and leads to high packet losses. We believe this high packet loss is the key reason behind the poor performance of Spray and Wait in this experiment.

In addition, LAROD-LoDiS performs better than Spray and Wait but worse than LER and LECAR. Although LAROD-LoDiS tries to keep the number of copies per packet low, it fails to do so from time to time, leading to congestion. Moreover, it lacks the location estimation mechanism that highly contributes to poor performance compared to LER and LECAR.

### 5.2. Performance Evaluation for the Hop Count per Packet

**Figure 12:**
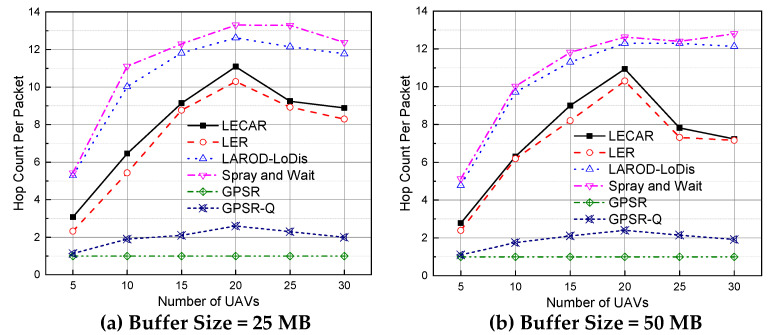
Performance comparison of the routing protocols regarding the hop count per packet when the buffer size is (**a**) 25 MB and (**b**) 50 MB.

We recorded the number of hops or UAVs that a packet travels from the sender to the destination. The average result is presented in Figure 12. A lower hop count is better because it requires fewer transmissions per packet. The GPSR has the lowest hop count indicating that it fails to route the packets successfully and only delivers when the destination is a one-hop neighbor. This finding explains the most deficient performance in terms of packet delivery ratio. The GPSR-Q also has similar results. The packets could travel at most three hops, which also explains the poor packet delivery ratio. In addition, LER performs better, and LECAR takes a few more hops than LER. The LECAR protocol takes extra hops to avoid congestion, thus avoiding packet loss. The result is a better packet delivery ratio than LER. LAROD-LoDiS and Spray and Wait take many more hops because they cannot take advantage of the path-planning mechanism and adequately direct the packets to the destination. Instead, they follow a broadcasting approach that leads the packets through a greater number of hops.

### 5.3. Performance Evaluation for the Number of Copies per Packet

**Figure 13:**
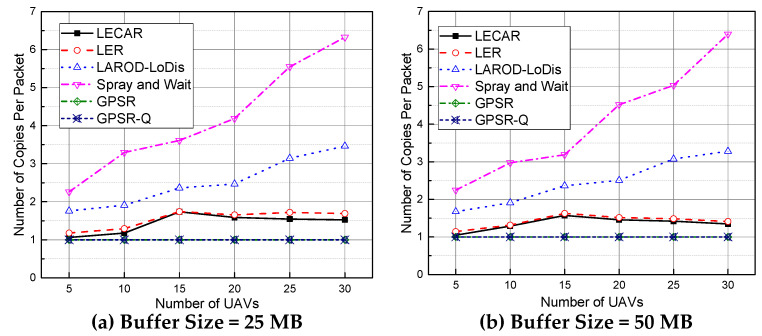
Performance comparison of the routing protocols regarding the number of copies per packet when the buffer size is (**a**) 25 MB and (**b**) 50 MB.

We calculated how many copies per packet simultaneously exist in the network during the entire experiment. Figure 13 presents the average result. The GPSR and GPSR-Q successfully keep only one packet at a time in the network due to their failure in successfully routing the packets, as discussed in previous subsections. Both LECAR and LER perform well by keeping the number of copies per packet at one in maximum cases and two in the worst cases due to the proper implementation of the ACK mechanism. The lack of such an ACK mechanism is the primary reason that LAROD-LoDiS results in a considerably larger number of copies per packet. Moreover, the highest result obtained by Spray and Wait in terms of the number of copies per packet is expected because it depends on the most increased data dissemination to route the packets successfully.

### 5.4. Performance Evaluation for the Number of Transmissions per Packet

**Figure 14:**
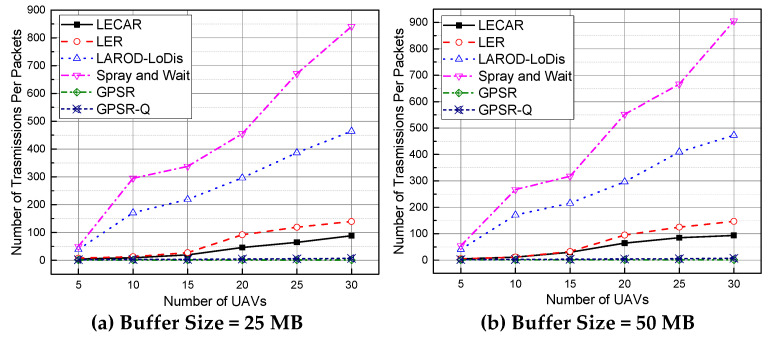
Performance comparison of the routing protocols regarding the number of transmissions per packet when the buffer size is (**a**) 25 MB and (**b**) 50 MB.

We recorded the number of transmissions that a packet experiences while traveling from the sender to the destination, and Figure 14 presents the average. Again, GPSR and GPSR-Q result in the lowest packet transmissions due to their failure in successful routing, as discussed. In addition, LECAR is the best performer due to its intelligent routing process. Moreover, LECAR keeps the hop count and the number of copies per packet comparatively low, resulting in fewer transmissions than LER, LAROD-LoDiS, and Spray and Wait. The previously described reason for the hop count and the number of copies per packet for LER, LAROD-LoDiS, and Spray and Wait applies as well.

### 5.5. Performance Evaluation for the Delay per Packet

**Figure 15:**
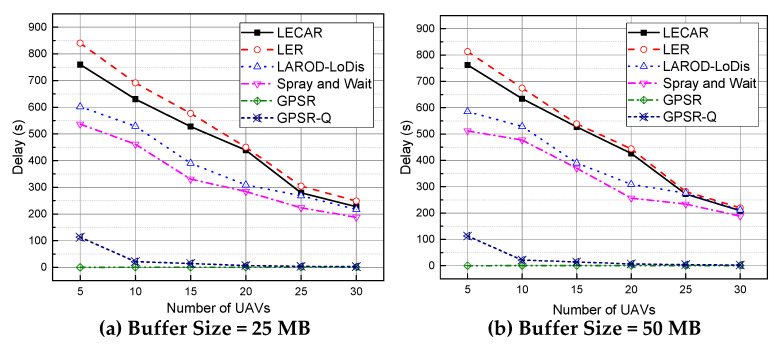
Performance comparison of the routing protocols regarding the delay per packet when the buffer size is (**a**) 25 MB and (**b**) 50 MB.

Figure 15 presents the delay experimented for the packets for the routing protocols in the simulation experiments. Both GPRS and GPRS-Q result in the lowest delay because they successfully transfer packets only when the destination is an immediate neighbor of the sender. Spray and Wait results in better performance due to its high data dissemination method. LAROD-LoDiS performed a bit worse than Spray and Wait but better than LECAR and LER. As previously observed, multiple copies of the same packet exist in the network, contributing to this better result. The relatively poor delivery ratio might also be a contributor. In addition, LECAR does not concentrate on quick packet delivery. Instead, it focuses on achieving the highest packet delivery using minimum energy. Hence, LECAR takes a comparatively long time to route the packet while avoiding congested paths and expending a minimum number of transmissions. However, when the number of UAVs increases, the delay drops significantly, resulting in a similar delay for Spray and Wait. This phenomenon is also evidence of the efficacy of LECAR. In contrast, LER takes a bit more time than LECAR because it follows congested paths from time to time due to its lack of awareness about the congestion situation.

### 5.6. Performance Evaluation for Overhead

**Figure 16:**
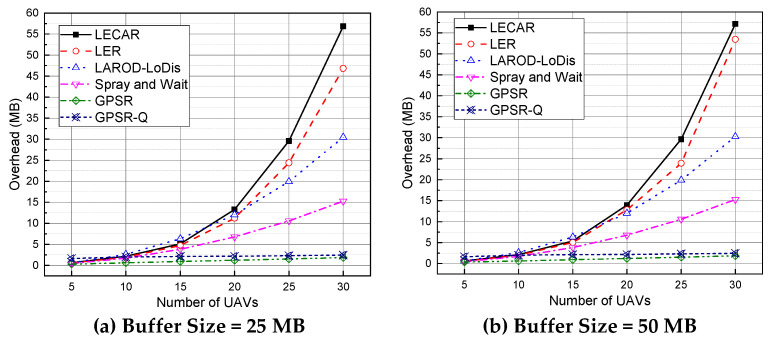
Performance comparison of the considered routing protocols regarding overhead when the buffer size is (**a**) 25 MB and (**b**) 50 MB.

Figure 16 presents the total overhead observed for the routing protocols in the experiments. The LECAR protocol generates the highest overhead compared with the others. It is because, LECAR must share the location information, buffer occupancy information, and other routing information to make the proper routing decision. The generated overhead is relatively low and almost the same as Spray and Wait and LAROD-LoDiS when there are 20 or fewer UAVs. Afterward, with the increase in the number of UAVs, the overhead increases significantly. The reason is understandable: LECAR is primarily designed for scenarios where a small number of UAVs cover a large area (i.e., when the density of the UAVs is relatively low). Therefore, the overhead of LECAR increases with the increase in the density of the UAVs. In addition, LER exhibits a similar tendency with slightly lower overhead than LECAR because it does not share the buffer occupancy information. Moreover, LAROD-LoDiS produces the next highest overhead because it shares the location information but in a briefer form. Spray and Wait generates moderate overhead. Finally, GPSR and GPSR-Q generate the lowest overhead following the previous results.

### 5.7. Performance Evaluation for Consumed Energy

**Figure 17:**
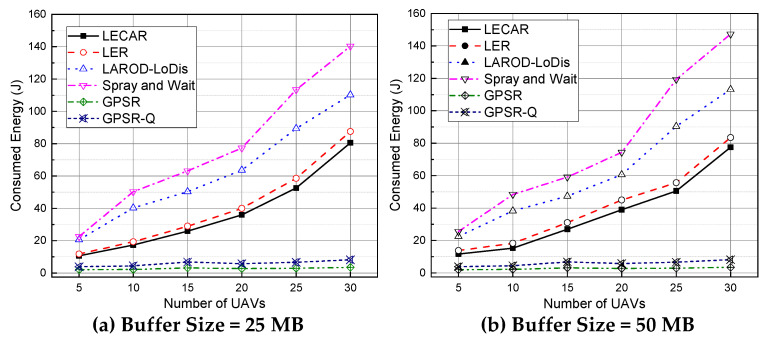
Performance comparison of the considered routing protocols regarding consumed energy consumption when the buffer size is (**a**) 25 MB and (**b**) 50 MB.

We recorded all transmissions (data and overhead) in the simulation experiments and calculated the total consumed energy during the experiments (Figure 17). We consider both data and overhead for proper evaluation of the performance concerning energy-efficiency. In DTN based approach, it is common that multiple copies of data packets can exist in the network that often cause extensive transmissions consuming significant energy. We can observe from Figure 17, GPRS and GPRS-Q consume the lowest energy. As explained earlier, GPSR and GPSR-Q often fail to forward the packet to the destination due to a lack of a proper mechanism to adapt in a sparsely populated network scenario. Additionally, they have the lowest overhead and packet delivery ratio. Thus, they result in the lowest transmission considering both data and overhead resulting in the lowest energy consumption. On the other hand, being the best performer in terms of packet delivery ratio, LECAR consumes the next lowest energy compared with LER, LAROD-LoDiS, and Spray and Wait. The low energy consumption by LECAR can be attributed to its small number of transmissions per data packet while keeping the number of packet copies at a minimum. The intelligent routing mechanism of LECAR allows it to avoid congested paths and routes towards the destination with each transmission. This primary factor helps LECAR overcome the extra overhead even in high-density scenarios. Similarly, the increased energy consumption by LER, LAROD-LoDiS, and Spray and Wait can be attributed to the increased number of transmissions per data packet along with the generated overhead.

## 6. Conclusions

In this work, we addressed the problem of balancing a high packet delivery ratio and low energy consumption for UAVs in a sparsely populated network scenario. To resolve the problem, we proposed LECAR, a new DTN-based routing protocol that takes advantage of the mobility model by estimating the location of the destination UAV and approximating the distance to the destination. Along with this estimated distance information, LECAR considers the congestion state of the neighboring UAVs and routes the packet to a UAV that moves closer to the destination and has enough space in its buffer.

In extensive simulation experiments, LECAR demonstrated a high packet delivery ratio (on average, 27% increase than Spray and Wait) and low energy consumption (on average, 42% decrease than Spray and Wait) compared to the considered routing protocols. Moreover, in maximum cases, LECAR could maintain a single copy per packet at a time in the network. It also ensured low hop counts for routing a packet (on average, 34% less than Spray and Wait). Although it generated a relatively large overhead, the number of transmissions per data packet outweighed the extra overhead and resulted in low energy consumption. These results reveal that LECAR better balances packet delivery ratio and energy consumption considering a sparsely populated FANET scenario. Although LECAR is designed considering a specific scenario and mobility model, the key idea can be easily extended and adapted to any other scenario or mobility model.

In future work, we plan to extend LECAR to significantly reduce the overhead even in a high-density network scenario. We further plan to improve LECAR for minimizing the delay in packet delivery, even for low-density scenarios.

## Figures and Tables

**Table 1:** Comparison of the features among the related major routing protocols for FANETs.

Features	Kuiper et al. [14]	Spyropoulos et al. [12]	Bujari et al. [15]	Arafat et al. [19]	Shi et al. [23]	Khelifi et al. [24]	Aadil et al. [25]	Proposed LECAR
Energy-efficient	✕	✕	✕	✕	✓	✓	✓	✓
Path prediction	✕	✕	✓	✓	✕	✕	✓	✓
Support for sparsely populated scenarios	✓	✓	✓	✓	✕	✕	✕	✓
Unicast	✕	✕	✕	✓	✕	✕	✕	✓
Single copy	✕	✕	✕	✓	✕	✕	✕	✓
Considers location information	✓	✕	✓	✓	✓	✓	✓	✓

**Table 2:** Key parameters in the simulation experiments in Network Simulator 3.26.

Parameter Name	Parameter Value
Observation area	10,000m × 10,000 m
Scan area for each UAV	400m × 400 m
UAV speed	55–70 m/s
Transmission range	800 m
Wireless standard	802.11 b
Number of UAVs	5–30
Number of targets	0–10
Simulation time	60 min
Packet size	524 KB

## Abbreviations

UAV	Unmanned aerial vehicle
DTN	Delay tolerant network
LECAR	Location estimation-based congestion-aware routing protocol
FANET	Flying ad doc network
MANET	Mobile ad hoc network
VANET	Vehicular ad hoc network
LADTR	Location-aided delay tolerant routing protocol
AODV	Ad hoc on-demand distance vector routing protocol
ACK	Acknowledgement
Spray and wait	“Sprays” a number of copies into the network, and then “waits” till one of these nodes meets the destination
LAROD-LoDiS	Location aware routing for delay tolerant network, enhanced with a location service, and location dissemination service
GPSR	Greedy perimeter stateless routing protocol
GPSR-Q	Greedy perimeter stateless routing with queueing functionality
LER	Location estimation-based routing protocol
**Math symbols**	
*T_pheromone_update_*	Update time of the pheromone value of a cell
*T_1_hop_update_*	Update time of the one-hop neighbor entry
*φ*	average buffer occupancy
TTL	Time to live
Curr_Cell_ID	Current cell ID
Nxt_Cell_ID	Next cell ID
*hello_interval*	Interval between consecutive hello messages
*T_loc_update_*	Update time of the last known location of an UAV
*n*	Number of waypoints
*t_passed_*	Passed time since the location information was updated
*t_s_*	Time a UAV needs to fly over a cell at the highest speed
*d_ij_*	Distance between two UAVs
(*x_i_*, *y_i_*, *z_i_*)	Coordinates of *UAV_i_*
(*x_j_*, *y_j_*, *z_j_*)	Coordinates of *UAV_j_*
$avg\_d_{n_{ij}d}$	Average distance from current and possible future location to destination
$F\_avg\_d_{n_{i}d}$	Final average distance considered the one- and two-hop neighbors
*α*	Constant with a value of 0.5
*Π*	Normalized distance
*φ*	average buffer occupancy
*φ_c_*	Current buffer occupancy
*B_current_*	Currently occupied buffer size
*B_total_*	Total buffer size
ω	Constant with a value of 0.6
*FC*	Forwarding cost
Ψ	Variable whose value depends on average buffer occupancy
